# In Vitro 3D Culture of Human Pathological Tendon Stem/Progenitor Cells Enables the Evaluation of Inflammatory Marker Modulation Induced by Semiconductor–Nanoparticle-Embedded Fabric

**DOI:** 10.3390/pharmaceutics18080907

**Published:** 2026-07-23

**Authors:** Adamo Lancellotti, Claudia Orlanno, Erwin Pavel Lamparelli, Federica Montella, Saveria Batti, Gina Myers, Nicola Maffulli, Giovanna Della Porta

**Affiliations:** 1 Department of Medicine, Surgery and Dentistry “Scuola Medica Salernitana”, University of Salerno, via S. Allende, 84081 Baronissi, SA, Italyelamparelli@unisa.it (E.P.L.); femontella@unisa.it (F.M.);; 2 Incrediwear Holdings, Inc., 3120 Thorntree, Chico, CA 95973, USA; 3 Department of Trauma and Orthopaedic Surgery, Faculty of Medicine and Psychology, Sapienza University, Via di Grottarossa 1035–1039, 00189 Rome, RM, Italy; nicola.maffulli@uniroma1.it; 4 School of Pharmacy and Bioengineering, Faculty of Medicine and Health Sciences, Keele University, Stoke-on-Trent ST5 5BG, UK; 5 Barts and the London School of Medicine and Dentistry, Centre for Sports and Exercise Medicine, Queen Mary University of London, Charterhouse Square, London EC1M 6BQ, UK; 6 Research Centre for Biomaterials BIONAM, University of Salerno, via Giovanni Paolo II, 84084 Fisciano, SA, Italy

**Keywords:** semiconductors and carbonized charcoal nanoparticles, embedded fabric, human tendon-derived stem/progenitor cells, 3D bioplotted environment, inflammation pathways

## Abstract

**Background/Objectives**: Human tendon stem/progenitor cells (hTSPCs) isolated from tendinopathic tendon tissue were used to identify key inflammatory biomarkers, including reactive oxygen species (ROS), pro-inflammatory cytokine release, and type III collagen expression. This cellular model was employed as an in vitro platform to investigate the effects of a semiconductor-nanoparticles embedded fabric (hereafter named fabric) on inflammatory marker regulation. **Methods**: hTSPCs were cultured in both conventional monolayer conditions and within a three-dimensional (3D) bioplotted methacrylated collagen (ColMA) scaffold under perfusion. The cells were exposed to a microenvironment enriched with negative ions and far-infrared radiation generated by fabric to assess its modulatory effects on native inflammatory and fibrotic pathways. **Results**: Fabric exposure significantly reduced ROS levels and modulated cytokine signaling pathways. These changes were associated with observed enhanced cell viability and proper extracellular matrix composition profile, characterized by reduced type III collagen and increased type I collagen deposition. Consistent findings were observed at both the protein and gene expression levels. Notably, these effects were more evident in the 3D culture system, likely due to its greater biomimetic relevance and ability to more accurately reproduce the native cellular microenvironment. **Conclusions**: Overall, these preliminary findings suggest that fabric promotes a transition from a fibrotic toward a more regenerative tendon-like phenotype, likely mediated by redox balance which probably improved ECM remodeling. Further studies should evaluate the potential of semiconductor-based fabrics to support tendon regeneration in vivo.

## 1. Introduction

Tendon homeostasis is a dynamic process maintained by the production of an extracellular matrix (ECM) by tenocytes and tendon stem/progenitor cells (TSPCs) [1,2]. Tendon architecture includes an extrinsic compartment composed of synovial sheaths (paratenon, epitenon, endotenon) with resident progenitor, immune, neural and vascular elements and an intrinsic core containing aligned collagen fiber bundles and resident cells (tenocytes, tenoblasts) within the ECM [3,4]. TSPCs reside within this milieu and express mesenchymal markers and exhibit three-lineage differentiation (adipogenic, osteogenic and chondrogenic). In a tenogenic environment, TSPCs express tendon-related genes such as Scleraxis-A (*SCX-A*), Decorin (*DCN*) and Tenascin-C (*TNC*) [5,6,7]. Human TSPCs derived from tendinopathic tissues exhibit marked phenotypic alterations, including loss of the typical fibroblast-like morphology and cytoskeletal disorganization [8]. These cells show reduced type I collagen expression, which is overtaken by increased type III collagen production. Additionally, they display elevated levels of ROS and a pronounced pro-inflammatory profile, characterized by increased secretion of cytokines such as interleukin (IL)-6, IL-10, and tumor necrosis factor α (TNF-α) [9]. 

Wearable and non-invasive devices have emerged as promising supportive therapies in orthopedics and rehabilitation [10]. Among these, functional fabrics incorporating carbonized charcoal and semiconductors have shown promising effects, first in sports medicine and later in the management of degenerative joint diseases and in post-surgical recovery [11,12]. Semiconductors and carbonized charcoal nanoparticle-embedded fabrics (hereafter referred to as “fabric”) are materials capable of releasing negative air ions (NAIs) and infrared (IR) radiation, both of which have been associated with biological effects. When they are heated above approximately 35 °C (or reach body temperature), a fraction of valence electrons migrates to the nanoparticle surfaces and interacts with surrounding ambient and, depending on the atmospheric composition and humidity, may generate a high concentration of negative air ions mixtures. Quantitative assessment of NAIs typically employs ion counters or spectrometers to measure spatial ion densities relative to the fabric surface and microenvironmental parameters [13,14,15,16]. At body temperature, the fabric emits also mid- and far-infrared (IR) radiation that can penetrate biological tissues and interact with structured water molecules and cellular components. These interactions may induce localized changes in blood and lymphatic circulation, potentially enhancing the delivery of oxygen and nutrients while facilitating the removal of metabolic waste [17,18,19]. Biological effects attributed to NAIs include reduction of ROS, suppression of endothelial nitric oxide synthase (eNOS) activity and reduction of pro-inflammatory cytokines, such as interleukin-1*α* (IL-1*α*), interleukin-1*β* (IL-1*β*) and interleukin-6 (IL-6). In endothelial cell in vitro models, exposure to those fabric has been associated with activation of anti-inflammatory pathways mediated by nitric oxide (NO) and calmodulin, with reduced expression of pro-inflammatory cytokines (e.g., IL-1β) and enzymes such as nitric oxide synthases (NOSs) and cyclooxygenase-2 (COX-2) [20]. Fabrics have also been reported to modulate in vitro key cellular functions, for example, stimulating ECM deposition by human adipose-derived mesenchymal stem cells (hADSCs) [21].

In chronic tendinopathy, persistent oxidative stress and inflammation are key upstream factors that impair tissue repair, disrupt extracellular matrix turnover, and progressively compromise tendon structure and function. Consequently, interventions with anti-inflammatory properties are of particular interest for tendon regeneration, as reducing the pathological microenvironment may help re-establish conditions favorable for matrix remodeling and maintenance of the tenogenic phenotype. TSPCs isolated from pathological tendon explants retain disease-associated characteristics and partially recapitulate the altered microenvironment observed in vivo. For this reason, they represent a valuable in vitro model for investigating the effects of fabric on pathological inflammatory biomarkers as well as matrix-related parameters involved in impaired tendon homeostasis of pathological environments [22]. 

On the other hand, traditional two-dimensional (2D) cell cultures, while widely used for their simplicity and reproducibility, impose artificial constraints on cells [23]. 3D culture systems offer, instead, a more physiologically relevant environment by promoting realistic cell–matrix interactions, supporting the establishment of nutrient and growth factor gradients, and enabling multidirectional cellular communication [24,25]. The integration of biomimetic scaffolds further enhances similarity to the native ECM [26], but when combined with dynamic culture conditions, such as perfusion or cyclic stretch bioreactors, these models more accurately replicate in vivo physiology, fostering cellular alignment, collagen deposition, and the mechanical maturation typical of tendon tissue [27,28].

The present study investigated the effects of semiconductor-and-carbonized-charcoal-nanoparticle-embedded fabric on TSPCs cultured under tenogenic conditions in both 2D and 3D environments. In particular, static 2D cultures and perfused 3D methacrylated-collagen-based constructs were maintained in the presence of growth differentiation factor-5 (GDF-5, 100 ng/mL) to support tenogenic differentiation [29]. Analyses were performed at multiple time points (7, 14, and 21 days) and included morphological, molecular, and functional assessments, such as ROS levels, cytokine expression, and the type I: type III collagen ratio, chosen as surrogate indicators of the inflammatory/fibrotic status of the pathological cells rather than as direct measures of tissue-level functional regeneration. The effects of fabric exposure were systematically evaluated across all conditions, considering and compared to control culture, selected as performed in the identical incubator without fabric.

## 2. Materials and Methods

### 2.1. Semiconductor-And-Carbonized-Charcoal-Embedded Fabric and NAI Concentration in the Incubator Environment

**Figure 1:**
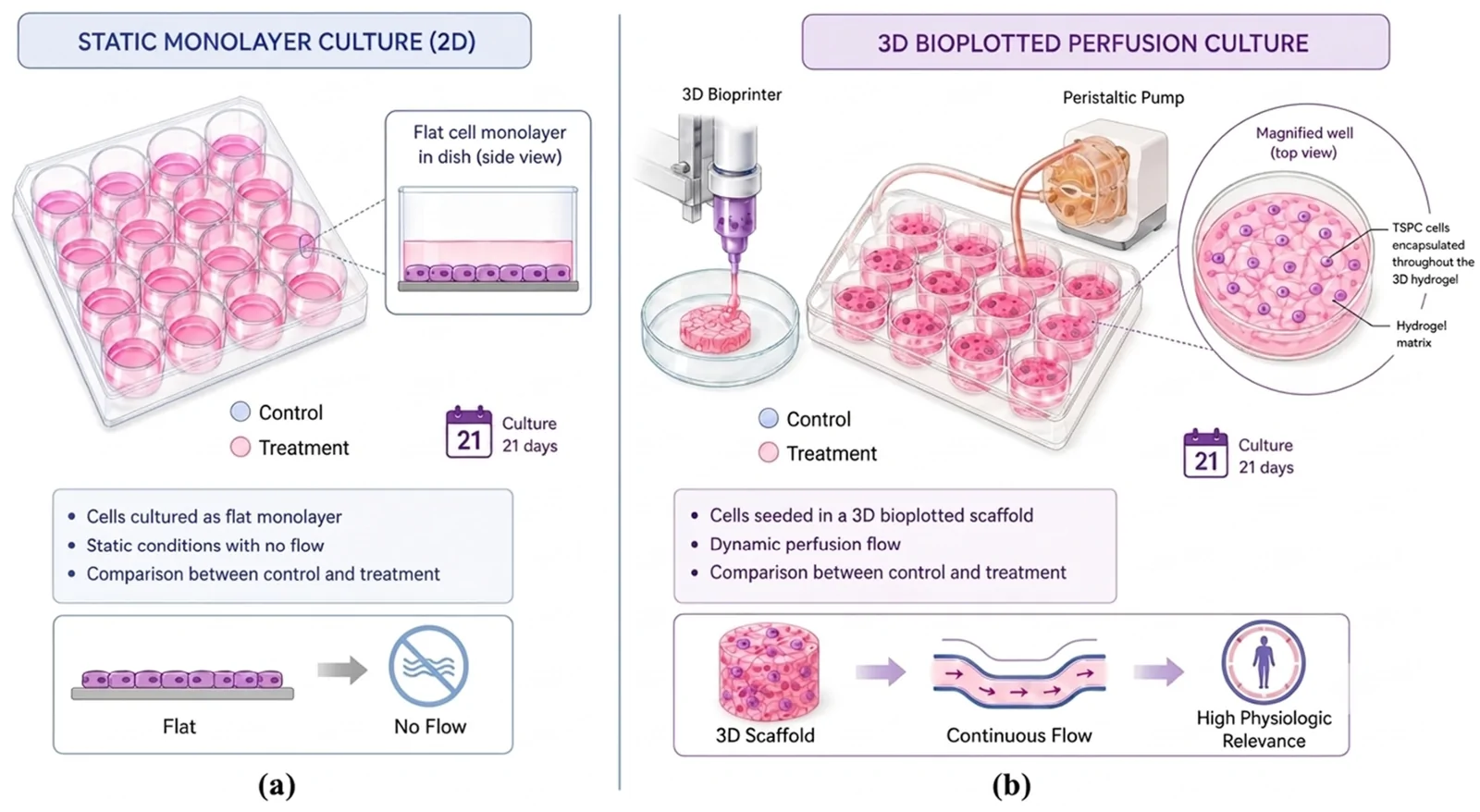
Schematic overview of experimental design used to evaluate the effects of fabric exposure on pathological hTSPCs. (**a**) static monolayer culture conditions (**b**) 3D bioplotted culture under perfusion were maintained for 21 days to check ROS levels and cytokine quantification. The image was generated with the free online version 5.5 of ChatGPT.

The same fabric (Incrediwear Inc., Chico, CA, USA) was used in all experiments at a fabric density of 4.1 × 10^−4^ g/cm^2^. The textile structure consisted of several braided fibers with an approximate thickness of 10 μm (see Figure 1). The fabric incorporates semiconductors and carbonized charcoal nanoparticles. When the fabric is thermally activated within a cell incubator, can ionize nearby air molecules, generating NAIs, including superoxide (O_2_^−^) and hydroxyl ions (OH^−^). This ionization process also creates a localized electrostatic field. The interconnected woven structure of the composite fabric provides a large surface area that enhances this thermally induced ion generation.

The surface morphology of the fabric was analyzed using a field-emission scanning electron microscope (FE-SEM; model LEO 1525, Carl Zeiss SMT AG, Oberkochen, Germany). Observations included untreated samples as well as samples subjected to autoclave treatment and a single 1 h UV irradiation cycle. Fabric specimens were cut into small pieces (5 × 5 mm) and mounted onto double-sided adhesive carbon tape attached to aluminum stubs. The mounted samples were vacuum-dried and then coated with a thin chromium layer (approximately 150 Å) using a turbo sputter coater (mod. K575X, EmiTech, Ashford, UK) prior to observation. Elemental composition of the fabrics was determined using an energy dispersive X-ray (EDX) analyzer (model INCA Energy 350, Oxford Instruments, Witney, UK) integrated with the SEM system.

Negative air ion concentrations were quantified within enclosed incubators (internal volume: 165,000 cm^3^) in the absence and presence of fabric (density: 4.1 × 10^−4^ g/cm^3^ relative to incubator volume). An average amount of fabric of 68.28 g/cm^3^ was applied in the whole incubator across all experiments, ensuring comparable ion exposure relative to cell number under both static and dynamic conditions. The amounts of ions in the incubator were measured by Air Ion Counter (mod. AIC3Pro, Alphalabinc.com, accessed on 1 January 2026). Fabric-containing incubators were used for the entire culture duration of each experiment, so that exposure was continuous over the selected time points. The present experimental design was intended to evaluate the biological effect of the complete fabric-conditioned culture environment; accordingly, NAIs were directly quantified, whereas FIR emission, although associated with fabric, was not directly measured within the incubator. For all experiments, control cultures were maintained in a second, identical incubator set under the same culture conditions but without the fabric.

### 2.2. Human Tendon Stem Cells Collected from Tendinopathic Surgery Explants of Achilles Tendon

Pathological tendon samples were obtained from the Achilles’ tendon of two tendinopathic male patient aged 53 and 61 years. Samples were obtained with prior informed consent according to protocols approved by the Institutional Review Board of “San Giovanni di Dio e Ruggi D’Aragona Hospital” (Salerno, Italy) (Review Board prot./SCCE n. 151 granted on 29 October 2020). Neither patient reported previous or concomitant tendon disorders. Human TSPCs were isolated from tendon biopsies using a previously validated protocol and expanded in minimum essential medium alpha (α-MEM, Corning Cellgro, Manassas, VA, USA) supplemented with 10% fetal bovine serum (FBS), 1% penicillin/streptomycin (Pen/Strep), and 1% L-analyl-L-glutamine (Corning^®^ GlutaGro^TM^, Corning, NY, USA) [30]. Cultures were maintained at 37 °C in a humidified atmosphere of 5% CO_2_. Cells at passage 2 were selected for all subsequent experiments. For control purposes, some experiments were also performed with healthy stem cells collected from the semitendinosus tendon of two male patients (42 and 55 years old) undergoing anterior cruciate ligament reconstruction. Healthy cells display a typical elongated and spindle-shaped morphology, consistent with a fibroblast-like phenotype; in contrast, pathological TSPCs exhibit a disorganized arrangement with a reduced alignment, indicative of altered cytoskeletal organization and loss of typical tendon-like features (see also Supplementary Figure S1) [31].

### 2.3. TPSC Culture Conditions: 2D Static vs. 3D Dynamic Systems

Human TSPCs were cultured under both 2D and 3D conditions for 21 days. In the 2D system, cells were maintained in a conventional monolayer using complete α-MEM supplemented with 10% fetal bovine serum (FBS), 1% penicillin/streptomycin, 1% L-glutamine, and 100 ng/mL growth differentiation factor-5 (GDF-5; PeproTech, Cranbury, NJ, USA). This formulation is referred to as a tenogenic medium, as GDF-5 promotes tenogenic differentiation through the upregulation of tendon-specific markers [23].

Bioplotting conditions were previously optimized and described elsewhere [9]. For 3D culture, constructs were prepared using 1.50 mL of methacrylated type I collagen (PhotoCol^®^, Advanced BioMatrix, Carlsbad, CA, USA), neutralized with 120 μL of neutralization solution (Advanced BioMatrix), and supplemented with 125 μL of lithium phenyl-2,4,6-trimethylbenzoylphosphinate (LAP) (Advanced Biomatrix, CA, USA) as a photoinitiator. A cell suspension was incorporated to achieve a final concentration of 2.5 × 10^6^ cells/mL. Scaffolds were then bioprinted using a BIO X bioprinter (CELLINK, Gothenburg, Sweden) according to previously optimized parameters (Table 1) [30].

Briefly, syringe and bed temperatures of the bioplotter were set to 20 °C and 40 °C, respectively, to ensure an initial gelling of ColMA during bioprinting and a proper set of the scaffold structure on the printing bed. The same tenogenic medium described for 2D cultures was used for 3D constructs, which were maintained under continuous perfusion (1 mL/min) within a custom-designed bioreactor able to ensure a proper mass transfer and metabolites exchanges, as described elsewhere [22]. Dynamic culture continuously recirculating culture medium in a closed-loop system, with weekly medium replacement. Further details are reported elsewhere [20].

### 2.4. Live and Dead Assay After Biofabrication

Cell viability within the 3D scaffolds was assessed using the Live/Dead kit (Cell Stain Double Staining kit, Sigma-Aldrich, Milan, Italy), according to the manufacturer’s instructions. Briefly, at 0, 7, 14, and 21 days of dynamic culture, scaffolds were collected, washed with 1× PBS (Corning Cellgro, Manassas, VA, USA) for 5 min at 37 °C, and then immersed in the working solution composed of 1× PBS with 0.2% (*v*/*v*) Calcein-AM solution (Sigma Aldrich, Milan, Italy) and 0.1% (*v*/*v*) propidium iodide solution (Sigma Aldrich, Milan, Italy) for 15 min at 37 °C. Following a triple wash with 1× PBS, fluorescence images were acquired at 10× magnification using a Nikon Eclipse Ti fluorescence microscope (Nikon Corporation, Tokyo, Japan).

### 2.5. ROS Quantification

2D culture was assessed by dichlorofluorescein (DCF) assay, which is well suited for standard adherent cell assays. The culture medium was removed and wells were washed with 100 μL of Hanks’ balanced salt solution (HBSS; GIBCO, Thermo Fisher Scientific, Waltham, MA, USA). Cells were then incubated with 100 μL of 5 µM 2’,7’-dichlorofluorescein diacetate (DCF-DA; Invitrogen^TM^, Thermo Fisher Scientific, MA, USA) in HBSS for 30 min at 37 °C and 5% CO_2_. After incubation, the DCF-DA solution was removed and wells were washed with 100 μL HBSS to remove excess unincorporated dye. Finally, 100 μL of HBSS was added to each well for fluorescence imaging. Fluorescence images were acquired using a Nikon Eclipse Ti fluorescence microscope (Nikon Corporation, Tokyo, Japan) and analyzed with ImageJ (version 1.54, National Institutes of Health, Bethesda, MD, USA) software.

3D culture was assessed by dihydroethidium (DHE) staining on tissue slices by the ROS Detection Cell-Based Assay Kit (DHE) (Cayman Chemical, Ann Arbor, MI, USA; Cat. No. 601290), as previously optimized in our laboratory. This assay is more suitable for 3D culture and allowed reliable detection while preserving spatial information within the bioprinted construct. Cryostatic sections were brought to room temperature, incubated at 37 °C for 20 min, and subsequently allowed to equilibrate at room temperature for 15 min. A hydrophobic barrier was drawn around each section using an ImmEdge Pen (Vector Laboratories, Newark, CA, USA). Sections were washed three times with the kit-provided assay buffer (5 min each) and then incubated with a dihydroethidium (DHE) probe (5 µM concentration) for 90 min at room temperature in the dark. Following incubation, sections were washed five times with assay buffer (5 min each) and mounted using an antifade aqueous mounting medium containing DAPI to counterstain cell nuclei. Image acquisition was performed with an inverted laser-scanning confocal microscope (TCS SP5; Leica Microsystems, Wetzlar, Germany) fitted with a Plan Apochromat 20×/0.70 NA dry objective; laser intensity, exposure time, and gain were kept constant across all samples and conditions. All data were normalized to Day 1 (24 h after bioprinting in the case of 3D culture).

### 2.6. Gene Expression

Both 2D- and 3D-derived pellets were resuspended in 350 µL of RNeasy Micro lysis buffer (Qiagen, Hilden, Germany) and RNA extraction was performed using the RNeasy Micro Kit (Qiagen, Hilden, Germany) following the manufacturer’s instructions. After extraction, RNA was quantified spectrophotometrically using a BioSpec-Nano system (Shimadzu Corporation, Kyoto, Japan). Proteins were recovered from the initial column flow-through and precipitated by adding four volumes of cold acetone (−20 °C), followed by incubation at −20 °C for 2 h. Samples were centrifuged at 5000 rpm for 20 min at 4 °C. Residual acetone was carefully removed, and pellets were ice-dried. Complementary DNA (cDNA) was synthesized from total RNA using the iScript^TM^ cDNA Synthesis Kit (Bio-Rad Laboratories, Hercules, CA, USA), according to the manufacturer’s instructions. For each reaction, up to 1 µg of RNA (quantified spectrophotometrically) was used in a final volume of 20 µL. Reverse transcription was performed in an Applied Biosystems 2720 thermal cycler (Thermo Fisher Scientific, Waltham, MA, USA). The resulting cDNA was stored at −20 °C. Quantitative reverse transcription polymerase chain reaction (qRT-PCR) was performed using the SsoAdvanced^TM^ Universal SYBR^®^ Green Supermix (Bio-Rad, Cat. No. 1725271, Hercules, CA, USA) with validated primers for Scleraxis (*SCX-A*), Decorin (*DCN*), Tenascin-C (*TNC*), Collagen type I alpha 1 chain (*COL1A1*), and Collagen type III alpha 1 chain (*COL3A1*) (Bio-Rad, Hercules, CA, USA). qRT-PCR reactions were performed using a LightCycler^®^ 480 II System (Roche, Basel, Switzerland). All samples were analyzed in triplicate, and gene expression levels were normalized to Glyceraldehyde-3-phosphate dehydrogenase (*GAPDH*; Bio-Rad, Hercules, CA, USA). Relative fold changes were calculated using the 2^−ΔΔCt^ method. Gene expression levels were normalized to those of TPSCs at Day 1 of culture.

### 2.7. Immunofluorescence Assay

Cells were fixed with 3.7% formaldehyde for 30 min at RT followed by permeabilization with 0.1% Triton X-100 (Sigma-Aldrich) for 5 min and blocking with 1% bovine serum albumin (BSA) (Sigma-Aldrich) for 1 h. For type I and type III collagen staining, cells were incubated overnight at 4 °C with a rabbit polyclonal anti-type I collagen antibody (1:200, Abcam, Cambridge, UK) and a mouse monoclonal anti-type III collagen antibody (1:100; Santa Cruz Biotechnology, Dallas, TX, USA). Following incubation with the primary antibody, cells were incubated for 1 h at room temperature (RT) with the Alexa FluorTM 488 goat–anti-rabbit IgG (1:500; Thermo Fisher Scientific, Waltham, MA, USA) and the Alexa FluorTM plus 594 goat–anti-mouse IgG (1:500; Thermo Fisher Scientific, Waltham, MA, USA) antibodies. Cell nuclei were stained with 4′,6-diamidino-2-phenylindole (DAPI) solution (1:1000) for 5 min.

3D culture scaffolds were washed three times with phosphate-buffered saline (PBS), fixed in 4% paraformaldehyde (PFA) for 2 h at room temperature, and cryoprotected in 30% (*w*/*v*) sucrose solution at 4 °C overnight. Scaffolds were then embedded in optimal cutting temperature (OCT) compound and sectioned into 20 µm thick slices using a CM1950 cryostat (Leica, Wetzlar, Germany) at −20 °C. Sections were collected onto SuperFrost Plus adhesion slides (Epredia, J1800AMNZ) and stored at −20 °C until further processing. Sections were permeabilized, as indicated before. For type I and type III collagen staining, sections were incubated overnight at 4 °C with a rabbit monoclonal anti-type I collagen antibody (1:200, EPR894-89, ab264074, Abcam, Cambridge, UK) and a mouse monoclonal anti-type III collagen antibody (1:200, FH-7a, ab6310, Abcam, Cambridge, UK) diluted in PBS + 1% BSA. Following three PBS washes (5 min each), sections were incubated for 1 h at room temperature with Alexa Fluor^TM^ 488 goat anti-rabbit IgG (H+L) (1:500, A11034, Thermo Fisher Scientific, Waltham, MA, USA) and Alexa Fluor^TM^ Plus 594 goat anti-mouse IgG (H+L) (1:500, A32742, Thermo Fisher Scientific) secondary antibodies diluted in PBS + 1% BSA. After three final PBS washes (5 min each), sections were mounted using an antifade aqueous mounting medium containing DAPI (Vectashield Mounting Medium with DAPI, Vector Laboratories) and coverslipped. Fluorescence images were collected on an inverted Leica TCS SP5 laser-scanning confocal microscope (Leica Microsystems, Wetzlar, Germany), under uniform acquisition conditions (laser intensity, exposure time, and gain).

### 2.8. Cytokine Assay

Culture media was collected at 7, 14, and 21 days from both 2D and 3D conditions and stored at −80 °C until analysis. Cytokine levels were quantified using the Human Cytokine Magnetic 10-Plex Panel (Invitrogen, Thermo Fisher Scientific, Vienna, Austria) at the protein level, following the manufacturer’s instructions. This assay enables the quantitative detection of GM-CSF, IFN-γ, IL-1β, IL-2, IL-4, IL-5, IL-6, IL-8, IL-10, and TNF-α. Beads were read on a Luminex ^®^^TM^ 200^TM^ instrument (Luminex Corp., Austin, TX, USA). A standard curve was generated using serial dilutions of the provided standards, run in duplicate. Samples were considered positive when cytokine concentrations exceeded the limit of detection specified by the manufacturer. Secreted cytokines were prioritized because the main endpoint of this analysis was the inflammatory phenotype effectively released into the extracellular microenvironment under Fabric exposure; cytokine gene expression was not included in the present exploratory design.

### 2.9. Statistical Analysis

Statistical analyses were performed on triplicate data (*n* = 3) using GraphPad Prism software (version 8.0 for Windows, LLC, San Diego, CA, USA). Data were expressed as mean ± standard deviation (SD). A two-way analysis of variance (ANOVA) was applied to evaluate the effect of two independent variables and their interaction with the measured outcomes. When appropriate, pairwise comparisons between groups were performed using Student’s *t*-test to assess differences in mean values. A *p* value < 0.05 was considered statistically significant across all analyses.

## 3. Results

### 3.1. Pathological TPSCs: Reason for the Selection

TPSCs exhibit an intrinsically altered morphology (Supplementary Figure S1) and a gene expression profile consistent with their pathological origin when cultured in a tenogenic environment (supplemented with GDF-5 at 100 ng/mL) [32]. This dysregulated expression pattern is evident in both conventional 2D monolayer cultures and 3D dynamic systems (see Supplementary Figures S2 and S3, fabric− and fabric +). In particular, cells display marked overexpression of COL3A1, a hallmark of tendon degeneration often associated with extracellular matrix (ECM) disorganization and reduced mechanical integrity. Furthermore, gene expression changes were more clearly pronounced in the 3D dynamic culture system, which appeared to better support phenotypic expression compared with monolayer and static culture. Pathological TSPCs also showed constitutive upregulation of IL-6, consistent with persistent activation of inflammatory signaling pathways. Collectively, these observations support the choice of these human primary cells as a relevant in vitro model for studying tendon inflammation and degeneration, as they inherently reproduce key disease-associated features without the need for artificially induced oxidative or inflammatory stress.

### 3.2. Culture in Incubator Environment with Fabric and NAI Values > 1000 Ions/cm^3^

Experiment rationale is described in Figure 1a,b. Under both 2D and 3D culture conditions, equal numbers of fabric-exposed cultures were consistently maintained in the incubator across all experimental setups to ensure comparable assays between fabric exposure and control culture. Among all data sets a similar number of cells was also adopted.

During culture, the mean concentration of NAIs was measured at values > 1400 ± 150 ions/cm^3^, whereas control conditions were assumed to have <80 ± 12 ions/cm^3^. Far-infrared (FIR) flux densities direct measurement within the incubator environment was not performed due to technical constraints, such as substantial background radiation. This makes difficult to distinguish the specific FIR emission attributable to fabric from the ambient thermal signal. Furthermore, high humidity levels can absorb and scatter infrared radiation, reducing measurement accuracy and reliability. Because NAIs were the only physical parameter directly quantified in the incubator atmosphere, they represent the main experimentally supported mechanism associated with this effect in the current study; however, a possible contribution of FIR cannot be formally excluded.

**Figure 2:**
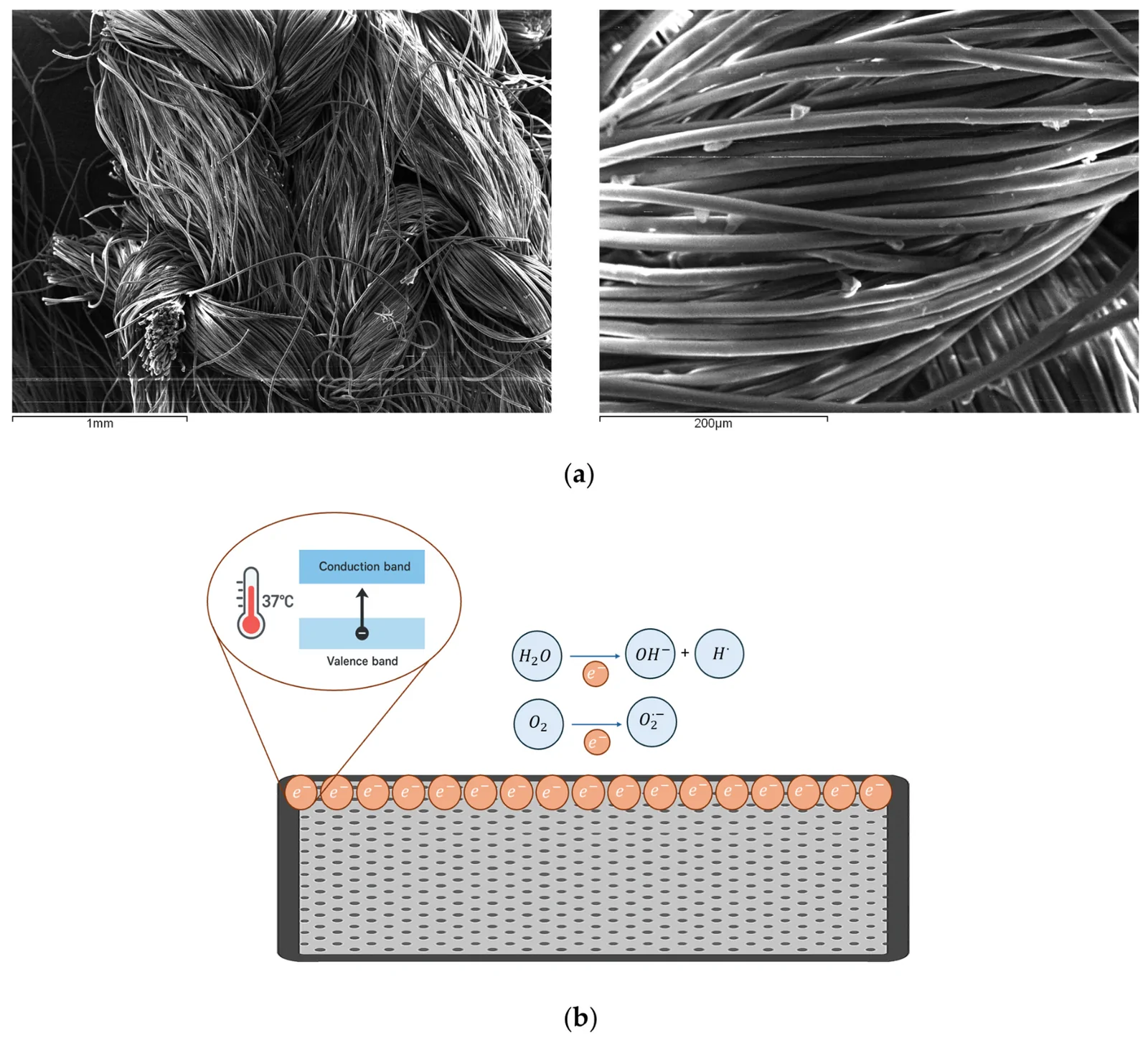
Fabric characterization and its negative air ion generation mechanism. (**a**) FE-SEM images of the fabric at two different magnifications, (**b**) schematic representation of the fabric illustrating its ion-producing mechanism.

The surface morphology and structural organization of the fabric are illustrated in Figure 2a, which present scanning electron microscopy (SEM) micrographs at different magnifications. The images reveal that the fabric textile is composed of multiple fibers with a mean diameter of approximately 10 μm, which are grouped into bundles (fasci) of roughly 100 fibers. These bundles are further intertwined through a braiding process, resulting in a complex, multiscale fibrous architecture that defines the overall textile structure. Figure 2b schematically illustrates the mechanism by which semiconductor nanoparticles ionize nearby air molecules, leading to the generation of negative air ions. 

Fabric also underwent both autoclave and UV sterilization: these did not induce any morphological alterations, with fiber integrity totally preserved. Further data are also reported elsewhere [20].

### 3.3. 2D Static Culture

**Figure 3:**
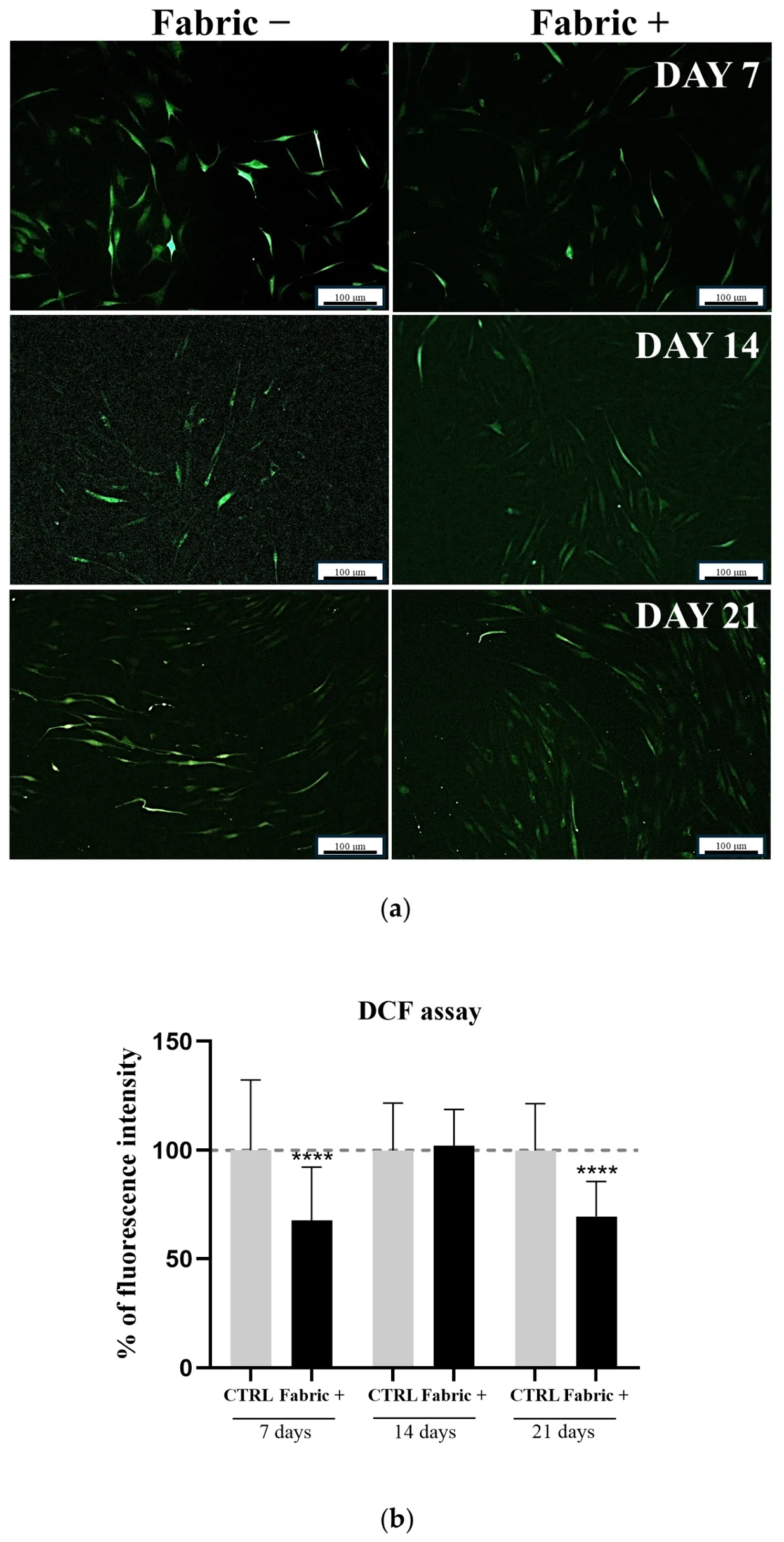
ROS production of 2D culture. (**a**) Fluorescence images of control (**left**) and fabric treated (**right**) culture after 7, 14 and 21 days. Magnification: 10×. Scale bar: 100 μm. (**b**) ImageJ analysis of DCF fluorescence intensity normalized to control cells and representing mean percentage ± standard deviation. **** *p* < 0.0001.

AS mentioned before, exogenous oxidative stress induction was not required, as tendinopathic TSPCs intrinsically exhibit elevated levels of reactive oxygen species (ROS), which are highly reactive molecules generated as by-products of cellular metabolism. This increased ROS production reflects a pre-existing oxidative imbalance associated with their pathological origin. Excessive ROS accumulation can disrupt cellular homeostasis by damaging proteins, lipids, and DNA, ultimately impairing cell function and viability. Accordingly, ROS levels were assessed to evaluate fabric potential capacity to mitigate oxidative stress (Figure 3a,b). Quantification of DCF fluorescence intensity was performed in 2D static cultures exposed to fabric and normalized to unexposed control cultures. A marked reduction in ROS levels was observed in fabric-treated cells compared with controls, suggesting the establishment of a more reductive, antioxidant-associated microenvironment.

**Figure 4:**
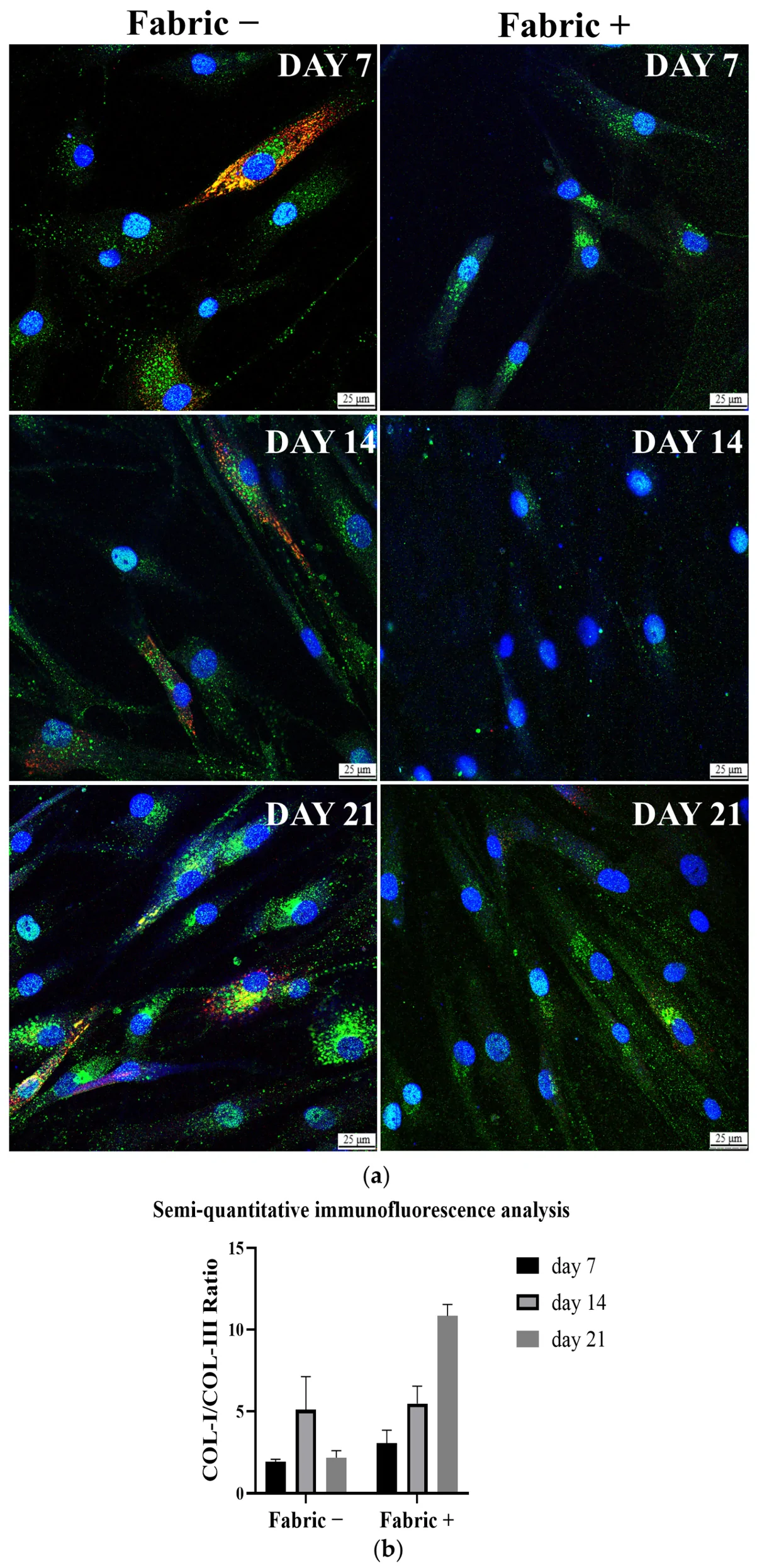
Immunofluorescence and quantitative analysis of type I and III collagen in TSPCs. (**a**) Immunofluorescence images of control and fabric treated along 7, 14, and 21 days of culture. Nuclei are stained in blue (DAPI), type I collagen in green, and type III collagen in red. Magnification: 63×. Scale bar: 25 µm. (**b**) Histogram showing the quantification of immunofluorescence (IF) signals for the type I:type III collagen ratio at 7, 14, 21 days under 2D static culture conditions with GDF-5-conditioned medium.

Confocal immunofluorescence images of the same 2D static cultures, stained for collagen type I and collagen type III in the presence or absence of fabric treatment, are shown in Figure 4a. The analysis was performed over a 21-day culture period to evaluate changes in extracellular matrix (ECM) composition and, in particular, the relative abundance of collagen type I and collagen type III, which are recognized markers of tendon tissue homeostasis or degeneration. Consistent with the pathological origin of the TSPCs used in this study, untreated cultures displayed a matrix profile characterized by relatively high collagen type III expression and limited collagen type I deposition. This pattern reflects the incomplete resolution of inflammation and the persistence of a degenerative ECM phenotype commonly observed in tendinopathic tissues. Therefore, the collagen I/III ratio was adopted as a relative indicator of matrix organization and/or tissue remodeling. It should be noted that this ratio was not intended to represent an absolute pathological threshold, as collagen expression levels may vary depending on donor characteristics, culture conditions, and analytical methodologies. However, it was adopted to better compare our results, through image-based fluorescence analysis.

We have observed that exposure to fabric progressively modified the ECM profile throughout the culture period. Qualitative examination of confocal images revealed a reduction in collagen type III signal intensity accompanied by increased deposition of collagen type I. Moreover, collagen type I fibers appeared more organized and uniformly distributed in fabric-treated cultures. Quantitative results confirmed the trends observed qualitatively, demonstrating a progressive increase in the ratio in response to fabric treatment (Figure 4b). The most pronounced differences were detected at day 21, when fabric-treated cultures exhibited a collagen type I/type III ratio exceeding 10, whereas untreated controls maintained values close to 2. These observations may suggest that fabric can atively promote a shift from a inflammatory ECM phenotype toward a more physiological matrix composition.

Cell nuclei, visualized by DAPI staining (blue), remained homogeneously distributed throughout the culture area, with no evidence of nuclear fragmentation, abnormal morphology, or reduced cell density. Interestingly, in monolayer static culture the effects of fabric on extracellular protein production appeared more pronounced than the overall cytokine modulation profile. While cytokine production provides important information regarding inflammatory activity, matrix synthesis and remodeling represent downstream functional outcomes that may be regulated through distinct pathways and temporal dynamics. However, further description of the cytokine profile and its implications will be reported in detail in the following.

### 3.4. 3D Dynamic Culture

**Figure 5:**
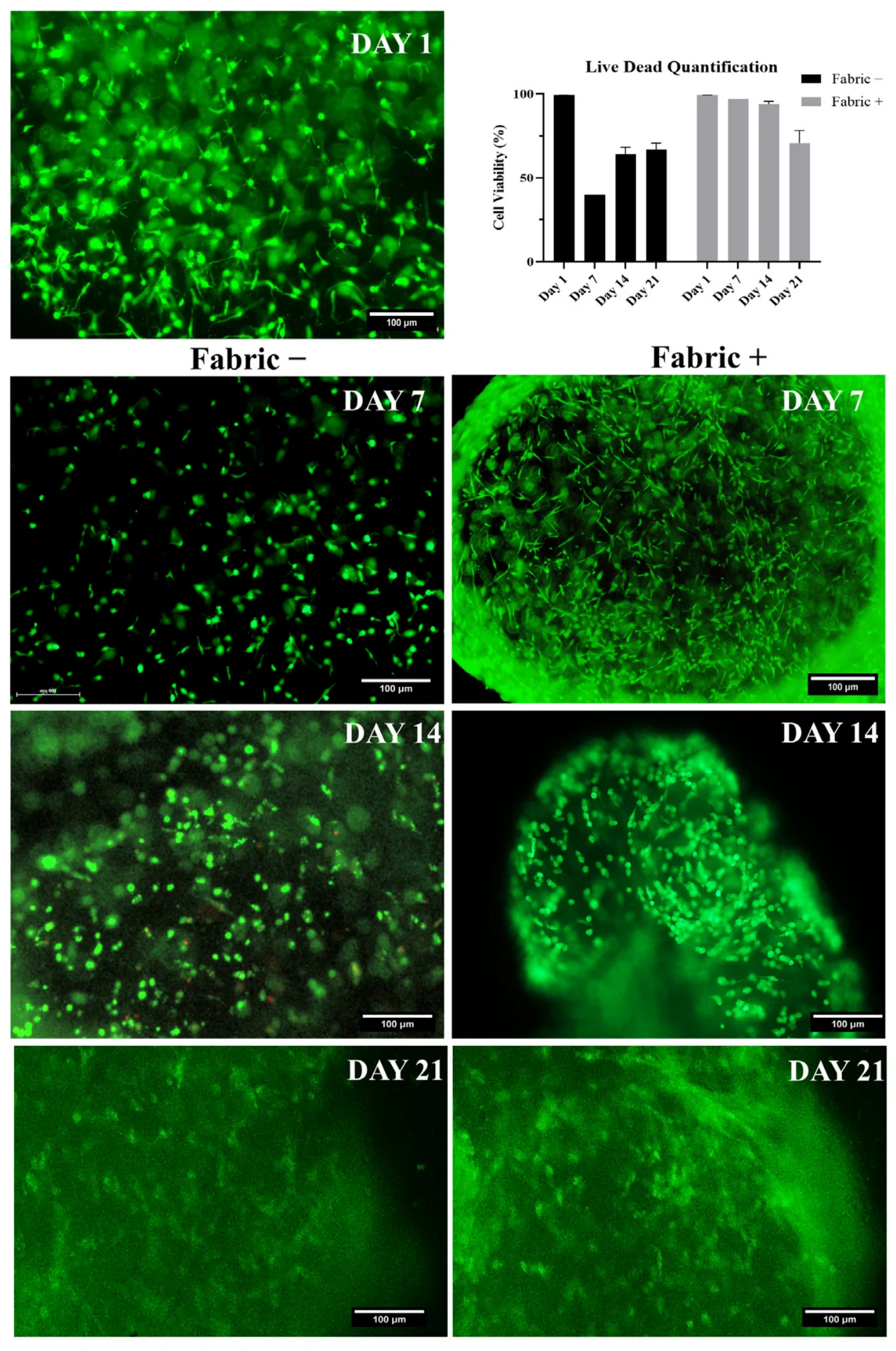
Live/Dead assay of 3D cultures. Representative fluorescence images showing live (green) and dead (red) cells in 3D cultures encapsulated within ColMa and maintained without (**left**) or with fabric (**right**) conditions from Day 7 to Day 21 of culture under perfusion. Data quantifications are reported as mean ± SD conditions at each time point in the plot (top right). Scale bar = 100 µm.

3D bioprinting was adopted to assemble 3D cultures. As summarized in Table 1, a needle size of 22 gauge was selected to ensure a controlled deposition of the scaffold structure with a fill density of 25% at a printing speed of 5 mm/s; about 1.5 × 10^6^ cell/mL were loaded and their viability at 99% was assessed immediately after bioprinting. Successful bioprinting was achieved under these conditions despite the known challenges associated with the printing of pathological primary cells, which are typically more sensitive and less robust than healthy cells during fabrication processes. Scaffolds (dimension: 5 mm × 2 mm) were printed and cultured under dynamic perfusion [33]. Notably, culture with fabric seemed to exhibit enhanced cell viability and proliferation, suggesting a positive effect on cellular metabolic activity (Figure 5). See also Supplementary Figure S4, with separated red/green channels.

**Figure 6:**
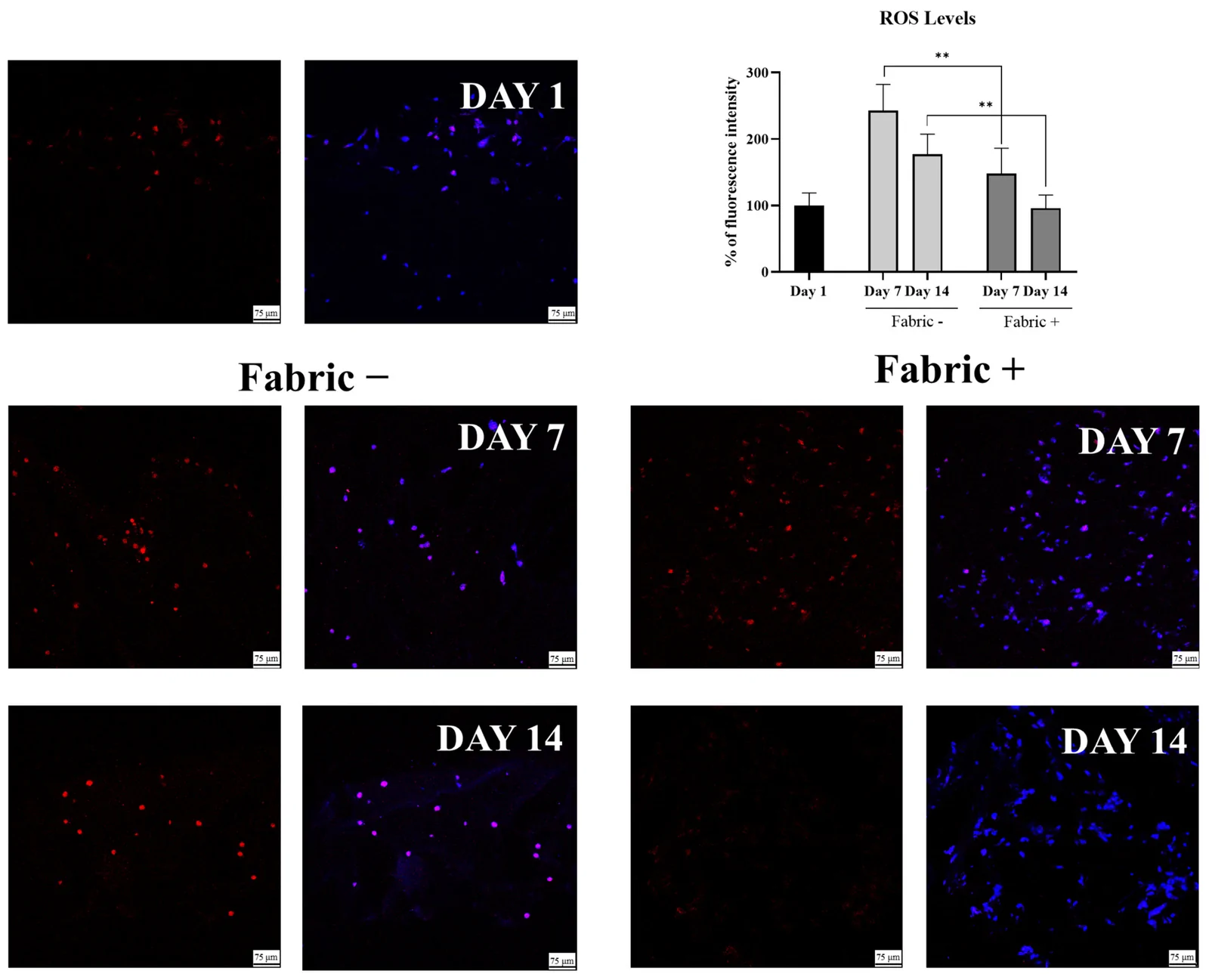
DHE assay for ROS detection in hTSPCs cultured in a 3D environment. Representative fluorescence images and quantitative analysis in 3D culture along Day 1, Day 7 and Day 14. Dihydroethidium (DHE) staining (red) indicates superoxide production, while nuclei are stained in blue (DAPI). Magnification: 20×. Quantification of fluorescence intensity is shown in the in the plot (top right). Data are presented as mean ± SD and normalized to Day 1 (100%). ** *p* < 0.01.

Within this environment, ROS levels increased during the culture period in both groups compared to the baseline at Day 1. This trend is compatible with the progressive metabolic demand and adaptation stress associated with long-term culture in a bioprinted and perfused 3D construct. Fabric-modified environment culture showed significantly lowered ROS levels at both Day 7 and Day 14 compared to the control group (*p* < 0.01). At Day 14, this effect was particularly evident; indeed, in the fabric treated group, ROS signals dropped significantly, reaching levels comparable to those measured at Day 1 (Figure 6). ROS levels were not assessed at Day 21, because the oxidative stress is an earlier time point biomarker, where it is known to play a more prominent role in driving the initial cellular response.

**Figure 7:**
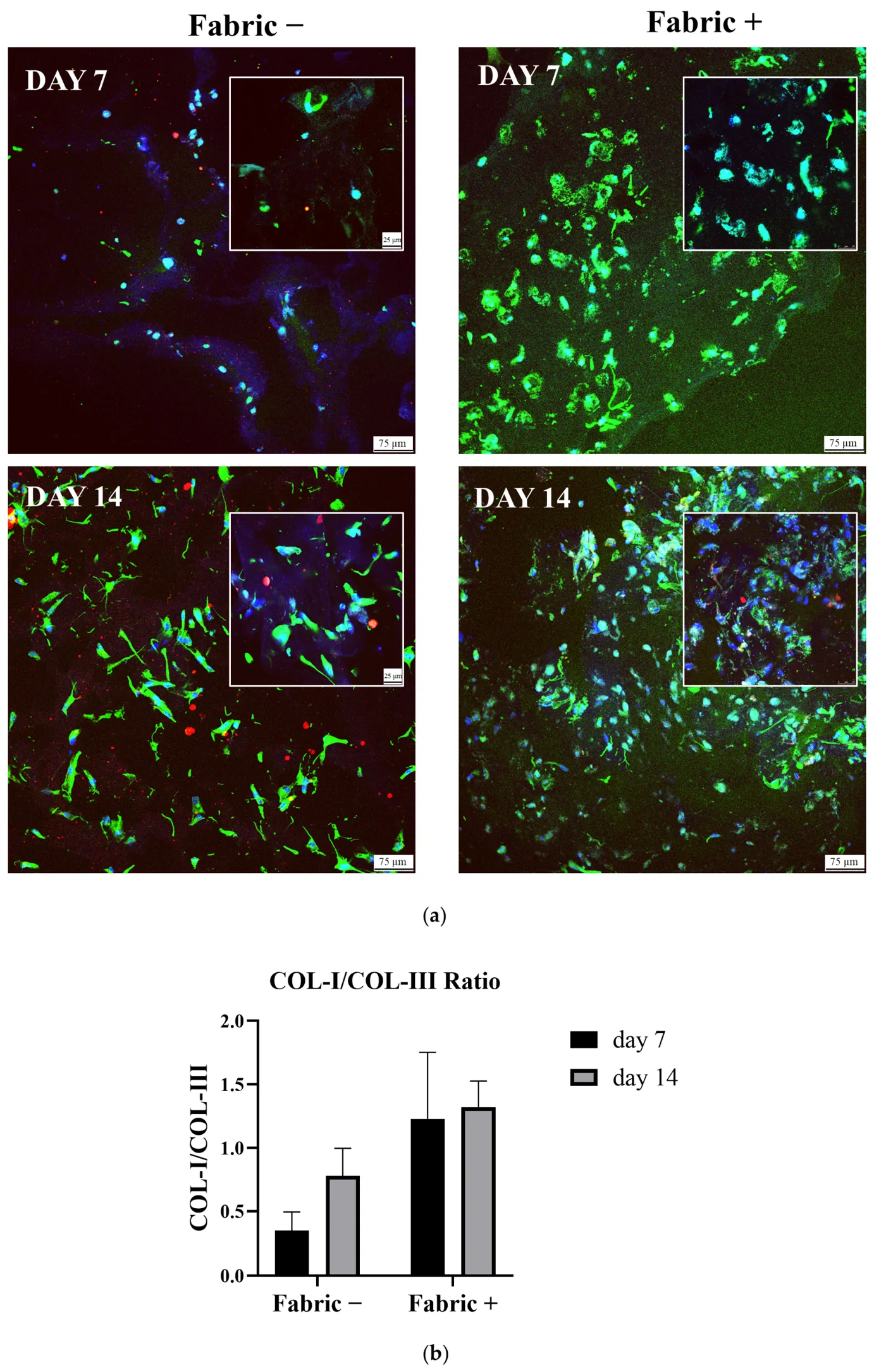
(**a**) Immunofluorescence analysis of collagen type I and type III deposition in ColMA 3D scaffolds at Day 7 and Day 14. Scaffold slices were stained for type I collagen and type III collagen at Day 7 and Day 14. Cell nuclei were counterstained with DAPI (blue). Magnification: 20× and 63×. Scale bar = 75 µm. (**b**) Histogram showing collagen type I:type III ratio quantified by IF signals at Days 7 and 14.

As for 2D culture, the 3D culture environment was tested by immunofluorescence to assess type I and type III collagen production in the culture for 14 days, in both control and fabric environments. Collagen I:III ratio was measured by quantitative analysis of fluorescence signals (Figure 7a,b). Control samples showed again a lower ratio value when compared to control cultures.

The difference between fabric + and fabric − samples was less pronounced in the 3D culture environment than in 2D cultures, with an increase in the collagen I/III ratio of approximately 0.7. This may be attributable to larger variability in sampling from 3D slices (with respect to 2D culture collection) or, alternatively, to the previously observed concomitant effect of the 3D culture system in promoting ECM protein production [9], which may partially mask the effect of fabric. However, these data are intended solely to reflect cellular activity and should not be interpreted as evidence of complete recovery of native tissue structure.

### 3.5. Cytokine Behavior

**Figure 8:**
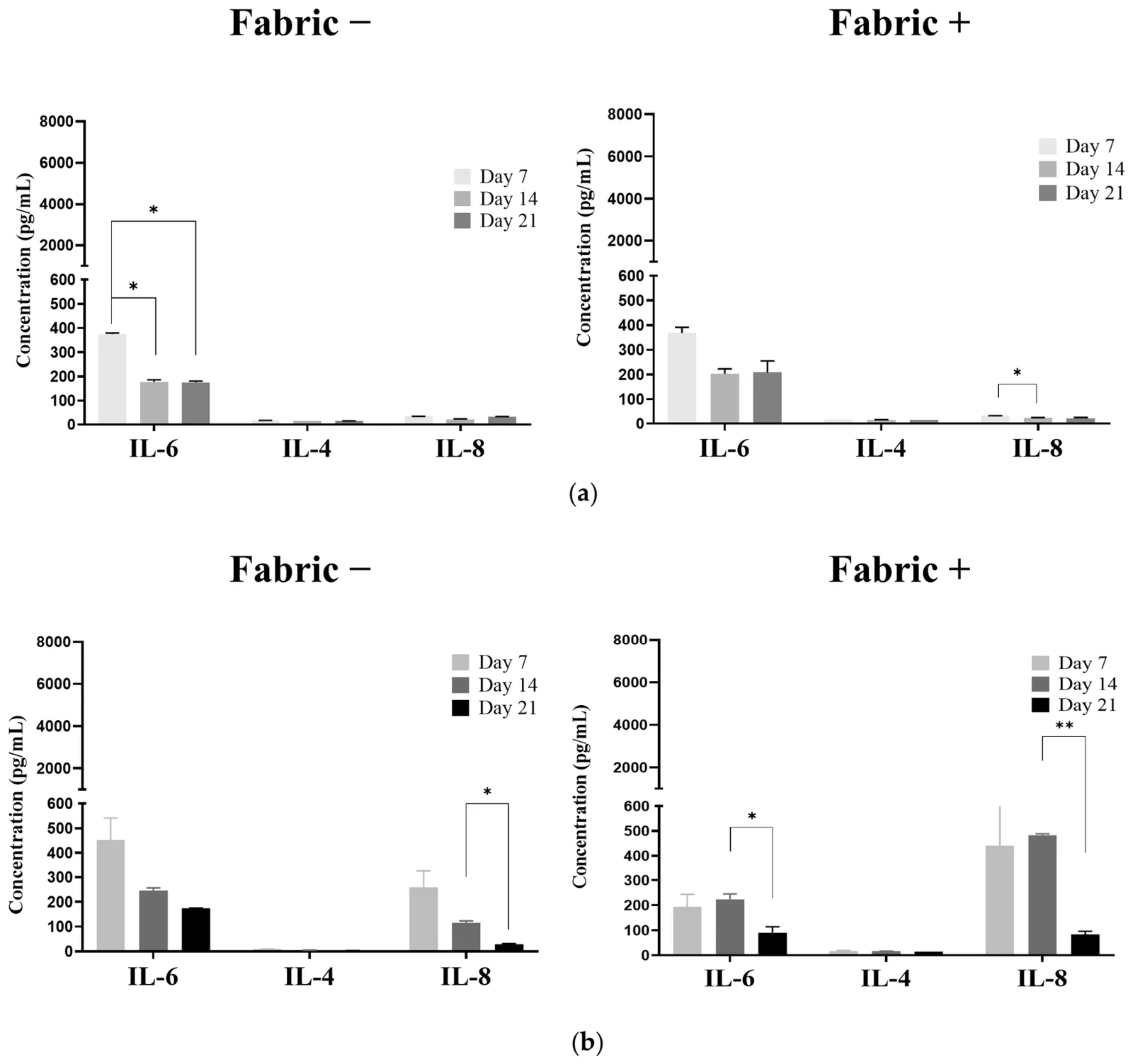
Cytokine profile of pathological hTSPCs cultured in 2D static vs. 3D dynamic layout. (**a**) Cytokine concentrations (pg/mL) of pathological hTSPCs cultured in static monolayer conditions and (**b**) in 3D dynamic culture with and without fabric for each experimental group. Data are presented as mean ± SD. Statistical significance is indicated (* *p* < 0.05; ** *p* < 0.01). See also Supplementary Figures S5 and S6.

The cytokine heatmaps of pathological hTSPCs cultured in monolayer conditions with and without fabric highlighted mainly IL-6 expression; however, the overall inflammatory profile was not significantly affected by fabric in these culture conditions (Figure 8a). Instead, the cytokine heatmap of 3D cultures revealed IL-6 levels significantly reduced in fabric-treated cultures compared to controls, with the strongest effect observed at the late time point (Day 21, *p* < 0.05). Conversely, IL-8 expression was consistently higher in the fabric-treated group across all time points. IL-4 levels showed a modest increase with fabric treatment, although this change was not statistically significant (Figure 8b).

The differences between 2D and 3D culture on cytokine profiles are likely related to the greater biological complexity of the 3D perfused system, in which cell–matrix interactions, spatial organization, and diffusion gradients collectively shape the secretory phenotype. Within this context, the persistence of IL-8 should be interpreted cautiously, as IL-8 may also contribute to remodeling-associated processes. By contrast, cytokine behavior in 2D cultures may reflect distinct time-dependent regulation of the inflammatory secretome; therefore, the stronger IL-6 modulation observed at Day 21 is biologically consistent with an earlier shift in redox status.

## 4. Discussion

The present study represents one of the first investigations into the biological effects of fabric on primary human cells isolated directly from pathological tendon tissue. Indeed, it is reported that semiconductor-and-carbonized-charcoal-nanoparticle-embedded fabric at a temperature above 32 degree Celsius emitted negative air ions (NAIs), which may reduce inflammation by scavenging reactive oxygen species (ROS) by signaling endothelial nitric oxide synthase (eNOS) activity and suppressing pro-inflammatory signaling pathways. The negative air ions (NAIs) may also enhance nitric oxide (NO) bioavailability to promote microvascular homeostasis [20]. On the other hand, tendon stem/progenitor cells (hTSPCs) collected from pathological tendon after its rupture intrinsically exhibit elevated oxidative stress, enhanced pro-inflammatory signaling, and an altered extracellular matrix (ECM) profile, making them a relevant in vitro model of chronic tendinopathy. Compared with healthy tendon tissue, which is predominantly composed of collagen type I and characterized by a highly organized fibrillar architecture, pathological tendons display increased collagen type III deposition [9]. This shift probably contributes to the formation of a mechanically inferior and disorganized matrix associated with persistent inflammation, impaired tissue function, and immune cell infiltration.

Given these disease-associated characteristics, pathological hTSPCs were selected to assess whether this bioactive fabric (in a given amount per volume and fixed number of cells) could constitutively modulate inflammatory pathways and influence matrix remodeling toward a more physiological tendon-like profile. To better approximate native tissue conditions, cells were cultured not only in conventional 2D monolayers but also within 3D bioprinted constructs maintained under continuous perfusion, and all data were always normalized with control experiments set without fabric in a second incubator [23].

The study specifically examined whether this fabric-conditioned environment could attenuate key hallmarks of tendinopathy, including oxidative imbalance and inflammatory dysregulation, which are closely linked to fibrotic-like ECM remodeling. Cultures were maintained in incubators either containing or lacking *fabric* (fixed amount of 4 × 10^−4^ g/cm^3^), generating a measured negative air ion (NAI)-enriched environment throughout the experimental period. Because NAI levels were directly measured whereas far-infrared (FIR) emission may be expected but not quantified, the current findings primarily support a mechanistic contribution of NAI exposure while recognizing that FIR radiation may also have influenced the observed cellular responses.

Overall, fabric exposure significantly reduced intracellular reactive oxygen species (ROS) levels, indicating a potential antioxidant effect. Since excessive ROS production is a hallmark of chronic tendon degeneration, mitigation of oxidative stress may represent an important upstream mechanism contributing to the biological benefits observed [32]. Importantly, ROS reduction should be also viewed as a regulatory event that may enable downstream normalization of inflammatory signaling and matrix synthesis rather than as direct evidence of tissue regeneration.

In addition to its effects on oxidative status, fabric appeared to influence cellular metabolism, including proliferative activity and ECM production. These changes were accompanied by a progressive shift toward a tendon-proper matrix composition. While similar trends were observed in 2D cultures, the 3D perfused model demonstrated more pronounced matrix maturation, characterized by increased COL1A1 expression, reduced COL3A1 expression, and enhanced organization of deposited collagen. Quantitative immunofluorescence further confirmed a higher collagen I:III ratio in fabric-treated constructs. Finally, the cytokine profile observed under 3D conditions also differed substantially from that detected in monolayer cultures, suggesting that the more physiologically relevant microenvironment may influence both inflammatory signaling and matrix remodeling.

The greater magnitude of fabric-associated effects observed in the 3D system should not be interpreted as evidence that 3D culture in vitro model exaggerates treatment responses. Rather, it likely reflects the ability of biomimetic and perfused culture conditions to preserve disease-relevant cellular behaviors and microenvironmental cues, thereby providing a more sensitive platform for detecting biological modulation. Similarly, the increase in the collagen I:III ratio should be regarded as an improvement relative to untreated pathological controls rather than as complete restoration of a healthy tendon phenotype. Establishing a universal physiological reference range remains challenging because collagen expression varies among donors and experimental conditions [25].

The divergent responses between 2D and 3D cultures are biologically plausible. Three-dimensional perfused constructs better retain the spatial organization, matrix interactions, and biochemical gradients that regulate oxidative adaptation and cytokine secretion in vivo. Consequently, both pathological features and treatment-associated changes become more evident within this context. The enhanced responsiveness observed under these conditions therefore reinforces the value of advanced 3D culture systems as informative models for studying tendon pathology and evaluating emerging therapeutic approaches. Across all experimental settings, the overall pattern of results was consistent with attenuation of inflammatory activity. The more pronounced responses detected in the 3D model further support its capacity to reproduce key aspects of the native tendon microenvironment and to reveal potential regenerative effects of fabric through relevant surrogate in vitro endpoints [22]. These findings also confirm the utility of ColMA scaffolds as advanced culture platforms capable of reproducing pathological processes while supporting tenogenic matrix formation within a modular and functionalized microenvironment [34].

Notably, modulation of collagen deposition was more substantial than changes in cytokine secretion, suggesting that fabric may act preferentially on pathways governing matrix production and tissue remodeling. This observation highlights the importance of evaluating both biochemical and structural outcomes when assessing the therapeutic potential of bioactive materials for tendon repair. The shift toward a more organized, collagen-I-rich matrix is particularly relevant because restoration of matrix quality is a prerequisite for functional tissue recovery [35].

The biological effects observed here are consistent with those reported for other emerging biophysical interventions, such as extracorporeal magneto-transduction therapy (EMTT), which has been shown to enhance tenocyte regenerative capacity and promote matrix remodeling through mechano-transductive mechanisms [36]. Progression toward a more regenerative and less scar-prone matrix architecture is considered essential for recovering tendon biomechanical competence [37]. From a clinical perspective, such structural improvements may ultimately improve tissue tolerance to progressive rehabilitation protocols and mechanical loading [38].

Several limitations should be considered when interpreting these findings. Although the 3D perfused constructs provide a more physiologically relevant platform than conventional monolayer cultures, they cannot fully replicate the complexity of the in vivo tendon environment, including systemic immune responses, vascular contributions, and neurohumoral regulation. Consequently, the reductions in ROS levels, changes in cytokine secretion, and increases in the COL1A1/COL3A1 ratio should be interpreted as indicators of a more favorable biological and matrix-remodeling profile rather than direct evidence of functional tendon regeneration. Future studies should incorporate additional functional endpoints, including cell migration, protein-level assessment of tenogenic differentiation, and biomechanical analyses, to better establish the translational relevance of these findings.

An additional limitation concerns the difficulty of accurately quantifying the local exposure of cells to negative air ions (NAIs) and far-infrared (FIR) radiation within the culture environment. The effective microenvironmental doses experienced by cells may differ substantially from those encountered in living tissues. Therefore, further investigation in appropriate animal models and subsequent clinical studies will be necessary to confirm the biological effects observed and determine their therapeutic significance.

## 5. Conclusions

The observed effects are consistent with the emerging concept that semiconductor-embedded fabrics can modulate cellular function through local physical phenomena, including the generation of negative air ions (NAIs) and the emission of far-infrared radiation. Although beneficial clinical outcomes associated with germanium-embedded fabrics have been reported, the biological mechanisms underlying their anti-inflammatory activity remain incompletely understood. In the present study, the NAI-enriched environment may have contributed to the observed responses by modulating redox homeostasis and attenuating inflammatory signaling pathways, potentially involving NF-κB-mediated mechanisms. However, the precise biochemical pathways responsible for these effects remain to be fully elucidated.

Nevertheless, the in vitro findings presented here support this fabric as a promising biophysical strategy for mitigating chronic inflammatory conditions. Furthermore, the hTSPC-based 2D and 3D culture platforms provided a robust experimental framework to investigate how modulation of oxidative stress, cytokine signaling, and extracellular matrix remodeling may collectively promote a more favorable tendon-like phenotype. Taken together, these results support an anti-inflammatory and pro-regenerative interpretation of fabric activity at the microenvironmental level. While they do not yet provide direct evidence of functional tendon repair or restoration of tissue biomechanics, they demonstrate the ability of fabric to influence key cellular and molecular processes that are relevant to healing and homeostasis.

## Figures and Tables

**Table 1:** Parameters used for 3D bioprinting of TSPCs.

Parameter	Value
Cell concentration	2.5 × 10^6^ cells/mL
Needle diameter	22 Gauge
Fill density	25%
Syringe temperature	20 °C
Bed temperature	40 °C
Printer speed	5 mm/s

## Data Availability

All data supporting the findings of this study are contained within the manuscript and its Supplementary Materials.

## Supplementary Materials

The following supporting information can be downloaded at: https://www.mdpi.com/article/10.3390/pharmaceutics18080907/s1, Figure S1: Brightfield morphology of healthy and pathological tendon stem/progenitor cells (TSPCs). Representative brightfield images of healthy (top) and pathological (bottom) TSPCs acquired at 4× magnification. Insets show higher-magnification views (10×) of representative areas. Scale bar = 200 µm; Figure S2: qRT-PCR analysis of key gene expression in pathological TPSCs cultured under 2D static versus 3D dynamic conditions in a standard incubator atmosphere. Expression levels of Tenascin-C (TNC), Collagen type I (COL1), and Collagen type III (COL3) were measured throughout the culture period. Data are presented as mean ± SD. * *p* < 0.05. Overall, the 3D dynamic culture environment appeared to better support gene expression compared to the 2D static; Figure S3: qRT-PCR analysis of key gene expression in pathological TPSCs cultured under 2D static versus 3D dynamic conditions in a incubator atmosphere with Fabric and NAI values > 1000 ions/cm^3^. Expression levels of Tenascin-C (TNC), Collagen type I (COL1), and Collagen type III (COL3) were measured throughout the culture period. * *p* < 0.05, **** *p* < 0.0001. 3D dynamic culture environment again appeared to better support gene expression compared to the 2D static. TNC showed a higher over expression in 3D environment throughout the culture period, reaching its highest expression on Day 21 (~11-fold), consistent with late-stage extracellular matrix maturation. COL1A1 increased at Days 14 and 21 (~25-fold), whereas COL3A1 peaked at Day 14 (~300-fold) and decreased by Day 21 (~90-fold); Figure S4: Live/Dead assay of 3D cultures in the absence or presence of Fabric**.** Representative fluorescence images showing live (green) and dead (red) cells in 3D cultures encapsulated within ColMa and maintained under fabric − (left) or fabric + (right) conditions from day 1 to day 21. Scale bar = 100X; Figure S5: Cytokine’s production of healthy hTSPCs in 2D static culture. (a) Heatmaps show temporal cytokine levels (Days 7, 14, 21). (b) Bar graphs display quantitative cytokine concentrations (pg/mL) at each time point. Data are presented as mean ± SD; Figure S6: Cytokine’s heatmaps related to Figure 8 of the manuscript.
