# Supplementary material for: In Vitro 3D Culture of Human Pathological Tendon Stem/Progenitor Cells Enables the Evaluation of Inflammatory Marker Modulation Induced by Semiconductor–Nanoparticle-Embedded Fabric
Source: Pharmaceutics. 2026 Jul 23;18(8):907. doi: 10.3390/pharmaceutics18080907 (PMC13516424; doi:10.3390/pharmaceutics18080907)
Supplement: Supplementary file 1 [file pharmaceutics-18-00907-s001.zip › pharmaceutics-4280389-supplementary.pdf]

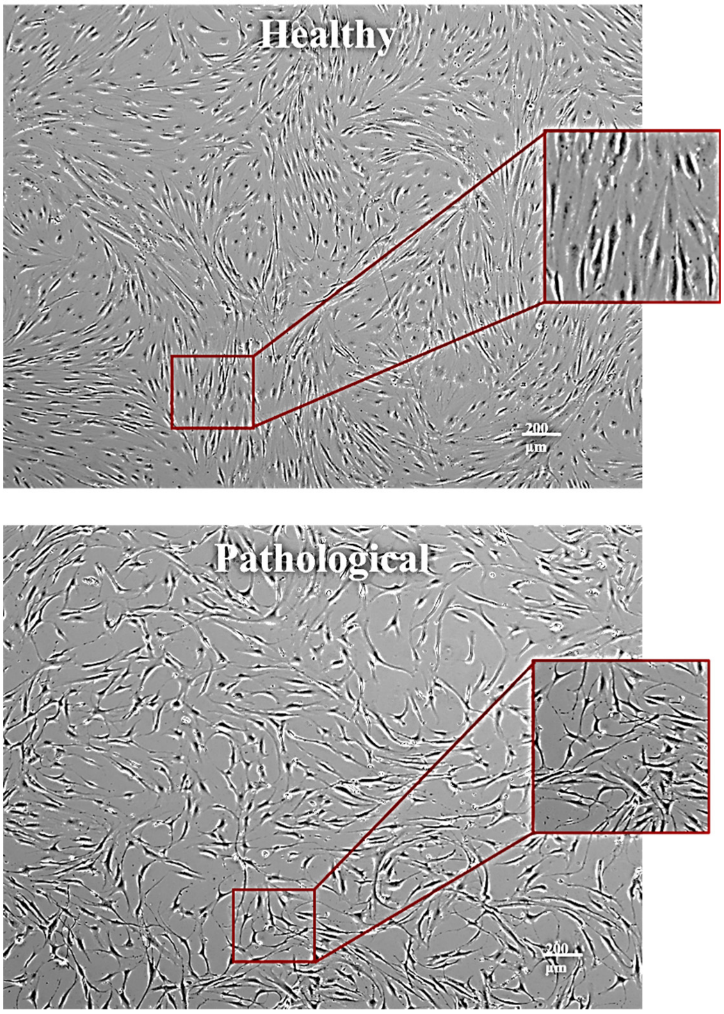

Supplementary Figure S1. Brightfield morphology of healthy and pathological tendon stem/progenitor cells (TSPCs). Representative brightfield images of healthy (top) and pathological (bottom) TSPCs acquired at 4× magnification. Insets show higher-magnification views (10×) of representative areas. Scale bar = 200 µm.

2D Fabric −

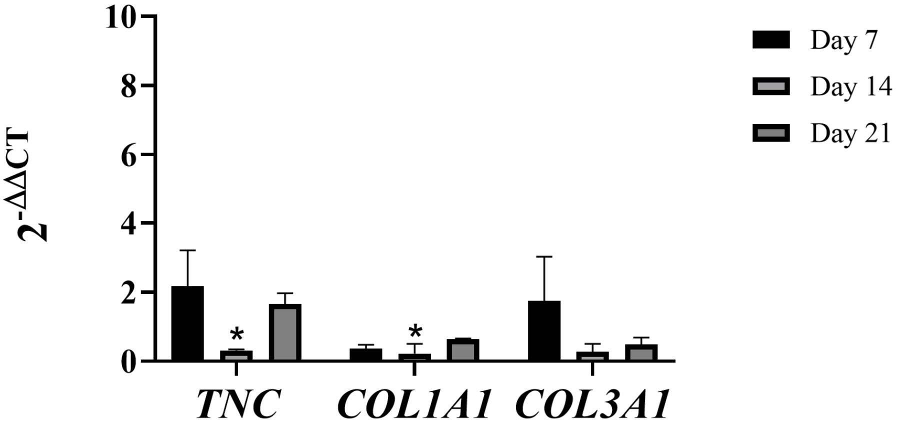

3D Fabric −

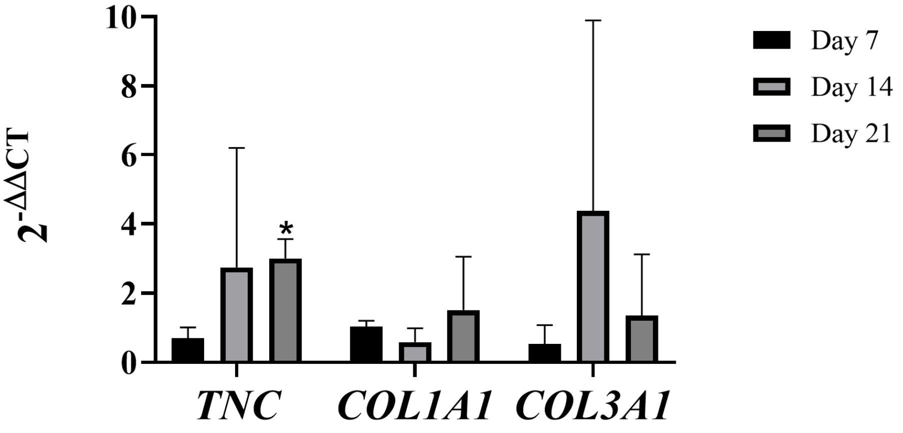

Supplementary Figure S2. qRT-PCR analysis of key gene expression in pathological TPSCs cultured under 2D static versus 3D dynamic conditions in a standard incubator atmosphere. Expression levels of Tenascin-C (TNC), Collagen type I (COL1), and Collagen type III (COL3) were measured throughout the culture period. Data are presented as mean ± SD. $*p < 0.05$. Overall, the 3D dynamic culture environment appeared to better support gene expression compared to the 2D static.

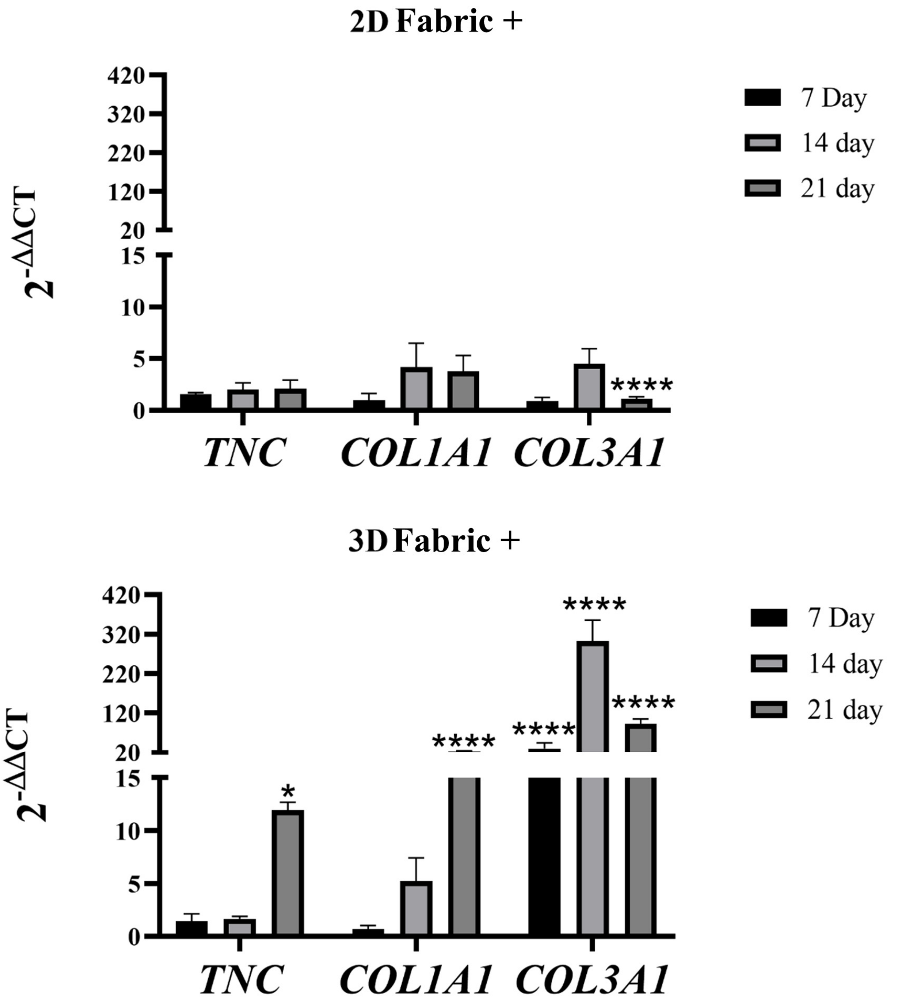

Supplementary Figure S3. qRT-PCR analysis of key gene expression in pathological TPSCs cultured under 2D static versus 3D dynamic conditions in a incubator atmosphere with Fabric and NAI values > 1000 ions/cm^3. Expression levels of Tenascin-C (TNC), Collagen type I (COL1), and Collagen type III (COL3) were measured throughout the culture period. * $p < 0.05$, **** $p < 0.0001$. 3D dynamic culture environment again appeared to better support gene expression compared to the 2D static. TNC showed a higher over expression in 3D environment throughout the culture period, reaching its highest expression on Day 21 (~11-fold), consistent with late-stage extracellular matrix maturation. COL1A1 increased at Days 14 and 21 (~25-fold), whereas COL3A1 peaked at Day 14 (~300-fold) and decreased by Day 21 (~90-fold).

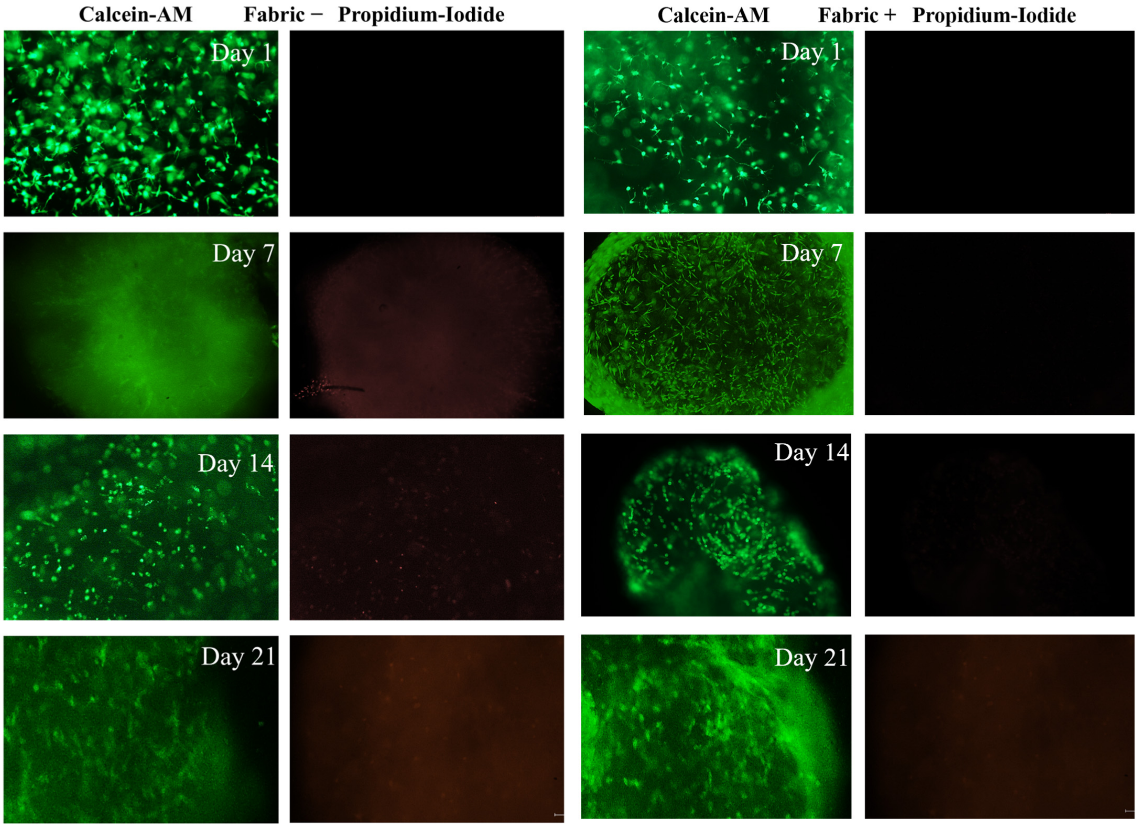

(a)

Supplementary Figure S4. Live/Dead assay of 3D cultures in the absence or presence of Fabric**.** Representative fluorescence images showing live (green) and dead (red) cells in 3D cultures encapsulated within ColMa and maintained under fabric −(left) or fabric + (right) conditions from day 1 to day 21. Scale bar = 100X.

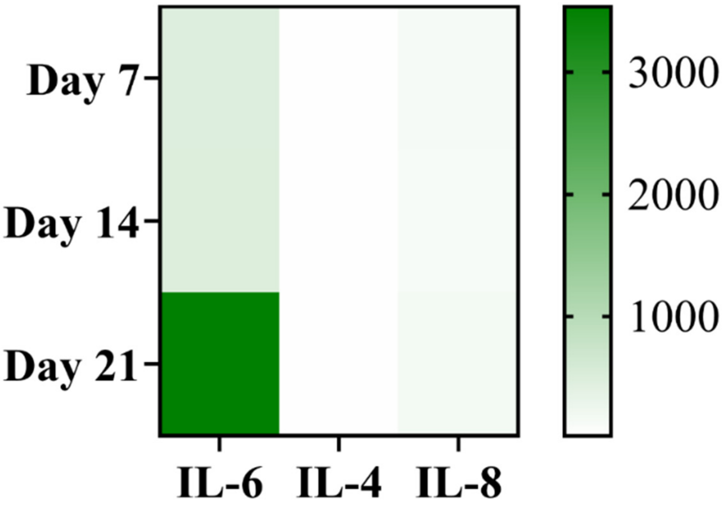

(a)

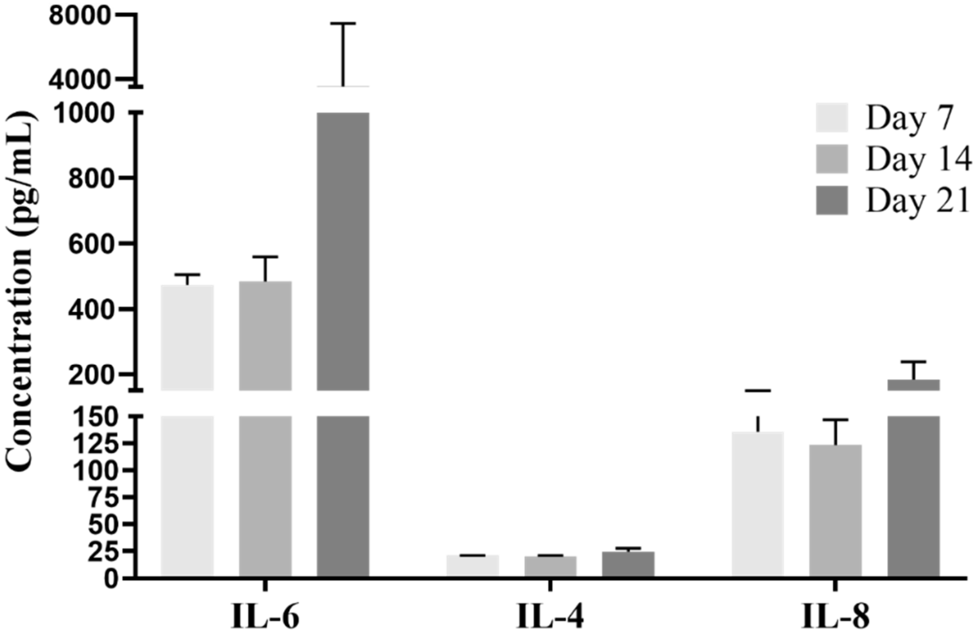

(b)

Supplementary Figure S5. Cytokine's production of healthy hTSPCs in 2D static culture. (**a**) Heatmaps show temporal cytokine levels (Days 7, 14, 21). (**b**) Bar graphs display quantitative cytokine concentrations (pg/mL) at each time point. Data are presented as mean ± SD.

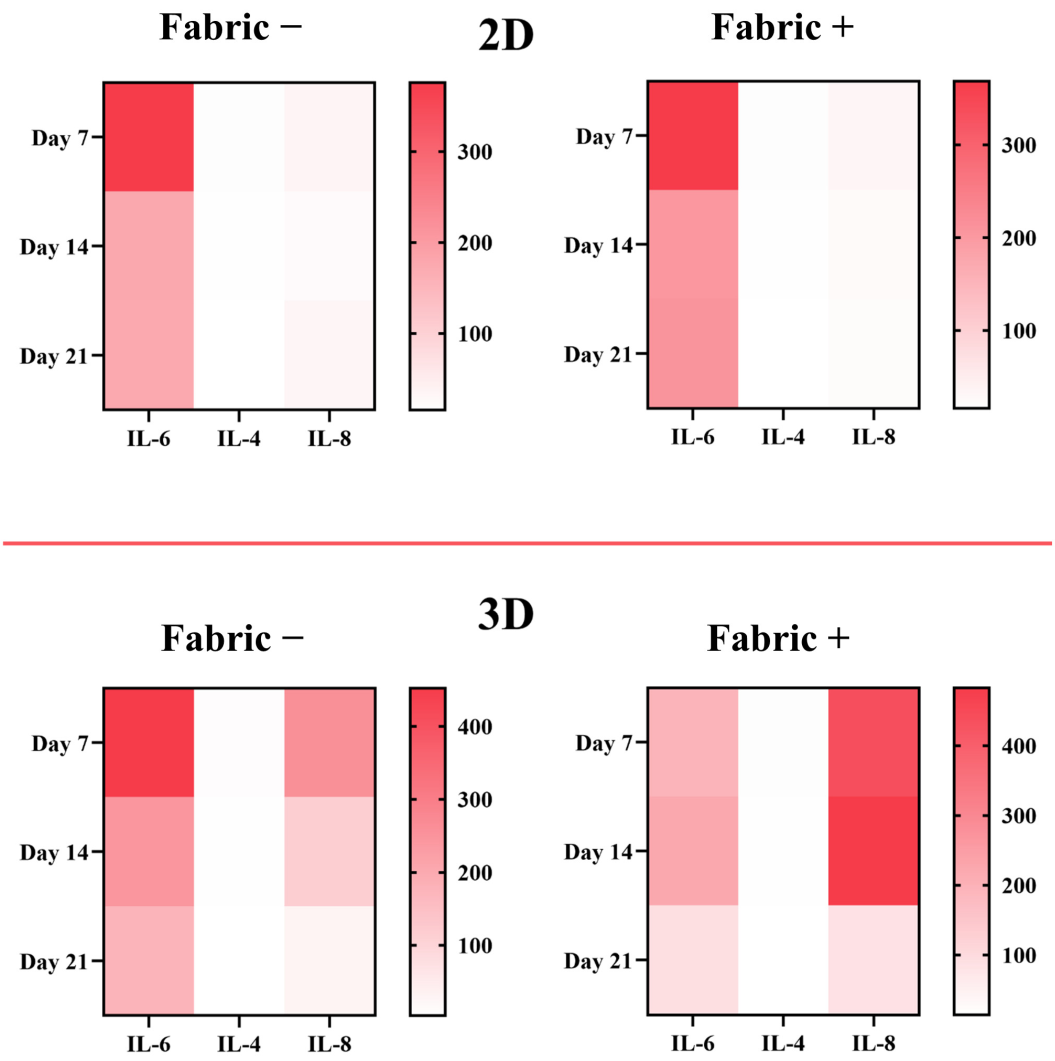

Supplementary Figure S6. Cytokine's heatmaps related to Figure 8 of the manuscript.
